# Supplementary material for: The kallikrein–kinin pathway as a mechanism for auto-control of brown adipose tissue activity
Source: Nat Commun. 2020 May 1;11:2132. doi: 10.1038/s41467-020-16009-x (PMC7195474; doi:10.1038/s41467-020-16009-x)
Supplement: Supplementary file 1 — Supplementary Information [file 41467_2020_16009_MOESM1_ESM.pdf]

Supplementary Information

The kallikrein-kinin pathway as a mechanism for auto-control of brown adipose tissue activity

Peyrou *et al.*

Supplementary Methods

Primary antibodies	Host	Provider	Catalogue number
UCP-1	Rabbit	Abcam (Cambridge, UK)	ab10983
KNG1	Rabbit	Abcam (Cambridge, UK)	ab175386
KNG2	Rabbit	AntibodyBcn (Barcelona, Spain)	-
TH	Rabbit	Millipore (Madrid, Spain)	ab152
β-actin	Mouse	Sigma Aldrich (Madrid, Spain)	#A5441

Secondary antibodies	Host	Provider	Catalogue number
HRP-conjugated anti-rabbit	Goat	Abcam (Cambridge, UK)	ab6721
HRP-conjugated anti-mouse	Goat	Bio-rad (Madrid, Spain)	#1721011
AlexaFluor 488-conjugated anti-rabbit	Goat	Thermo Fisher Scientific	A-11034

Reagents, chemicals and Kits	Provider	Catalogue number
Nucleospin RNA	Macherey-Nagel (Düren, Germany)	740955.250
High capacity RNA-to-cDNA kit	Thermo Fisher Scientific (Barcelona, Spain)	4387406
Taqman Master mix	Thermo Fisher Scientific (Barcelona, Spain)	11743-500
SYBRGreen Master mix	Thermo Fisher Scientific (Barcelona, Spain)	4472908
PVDF membranes	Amersham (Barcelona, Spain)	10600023
ECL	Millipore (Madrid, Spain)	WBKLS0500
Osmotic mini-pump	Alzet (Cupertino, CA, USA)	1007D
B1 antagonist R715	Tocris (Madrid, Spain)	3407/1
B2 antagonist HOE 140	Tocris (Madrid, Spain)	3014
Isoflurane	Zoetis (Madrid, Spain)	571329.8
dibutyryl-cAMP	Sigma Aldrich (Madrid, Spain)	D0627

Norepinephrine	Sigma Aldrich (Madrid, Spain)	A0937
CL 316,243	Sigma Aldrich (Madrid, Spain)	C5976
Bradykinin acetate salt	Sigma Aldrich (Madrid, Spain)	B3259
HMWK mouse recombinant protein	R&D system (Abingdon, UK)	RYD-2206-PI-010
Dapi	Sigma Aldrich (Madrid, Spain)	10236276001
Rosiglitazone	Sigma Aldrich (Madrid, Spain)	R2408
PepTag® Non-Radioactive Protein Kinase Assay	Promega (Madrid, Spain)	V5340
9-Plex Multi-Pathway Total Magnetic Bead Kit 96-well Plate Cell Signaling Multiplex Assay	Millipore (Madrid, Spain)	48-680MAG
9-Plex Multi-Pathway Total Magnetic Bead Kit 96-well Plate Cell Signaling Multiplex Assay	Millipore (Madrid, Spain)	48-681MAG
Pertussis Toxin	Tocris (Madrid, Spain)	3097
YM-254890	Wako (Madrid, Spain)	257-00631
Free glycerol reagent	Sigma Aldrich (Madrid, Spain)	F6428
Glycerol standard solution	Sigma Aldrich (Madrid, Spain)	G7793

**Taqman probes**	**Catalogue number**	**Gene Name**	**Catalogue number**
Ucp1	Mm00494069_m1	*Angiopt1*	Mm00456503_m1
Dio2	Mm00515664_m1	*Cd31*	Mm01242584_m1
Prdm16	Mm00712556_m1	*Flt1*	Mm00438980_m1
Ppargc1a	Mm00447183_m1	*Kdr*	Mm01222421_m1
Cebpb	Mm00843434_s1	*Vegfa*	Mm01281449_m1
Pparg	Mm00440945_m1	*Mrc1*	Mm00485148_m1
Lpl	Mm00434764_m1	*B1*	Mm04207315_s1
Glut1	Mm00441480_m1	*B2*	Mm00437788_s1
Glut4	Mm00436615_m1	*Fabp4*	Mm00445880_m1
Fas	Mm00494074_m1	*Ccl2*	Mm00441242_m1
Cd36	Mm01135198_m1	*Clec10a*	Mm00546124_m1

Dgat	Mm00515643_m1	*Nos2*	Mm00440502_m1
Lcad	Mm00599660_m1	*Acox1*	Mm00443579_m1
Mcad	Mm00431611_m1	*Leptin*	Mm00434759_m1
Adipoq	Mm00456425_m1	*Cidea*	Mm00432554_m1
Klkb1	Mm00434658_m1	*Klk1*	Mm00834006_g1
Ace	Mm00593654_cn	*Kng2*	Rn00754976_m1
Arg1	Mm00475988_m1	*Kng1/1*	Rn00754953_m1
Il6	Mm00446191_m1	*Kng1*	Rn01774060_m1
Tnf	Mm00443258_m1		
**Reference gene**	**Catalogue number**	**Reference gene**	**Catalogue number**
18S	Hs99999901_s1	*Cyclophilin*	Mm02342430_m1

**SybrGreen**	**Forward**	**Reverse**
Kng1	5’-CCTGGGGTAAAAAGTGGCAA-3’	5’-GGAGTAAAAGGTTGGAGAGCC-3’
Kng2 Hmwk	5’-GACTGCCCAGAGAACAGAACC-3’	5’-CCTCTCCTGTATCTGTGTAGA-3’
Kng2 Lmwk	5’-GCAGGAACAACTAGGCTCCTA-3’	5’-CATCTCAGGATTCTTCTGCTCC-3’
**Reference gene**	**Forward**	**Reverse**
Rps9	5’-GACCAGGAGCTAAAGTTGATTGGA-3’	5’-TCTTGGCCAGGGTAAACTTGA-3’

Scheme of primer design sites:

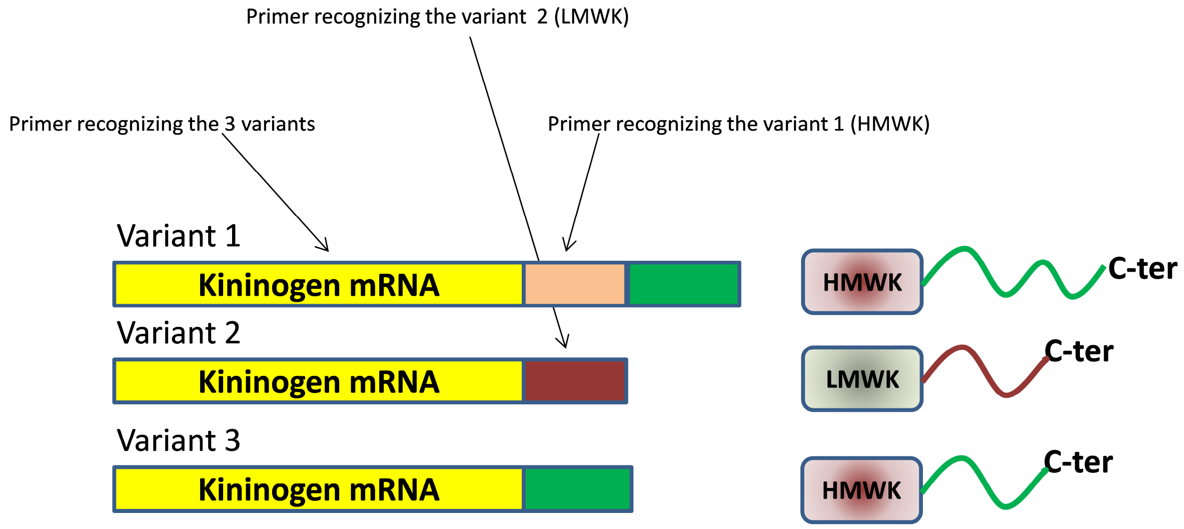

Supplementary Figures

Supplementary Figure 1

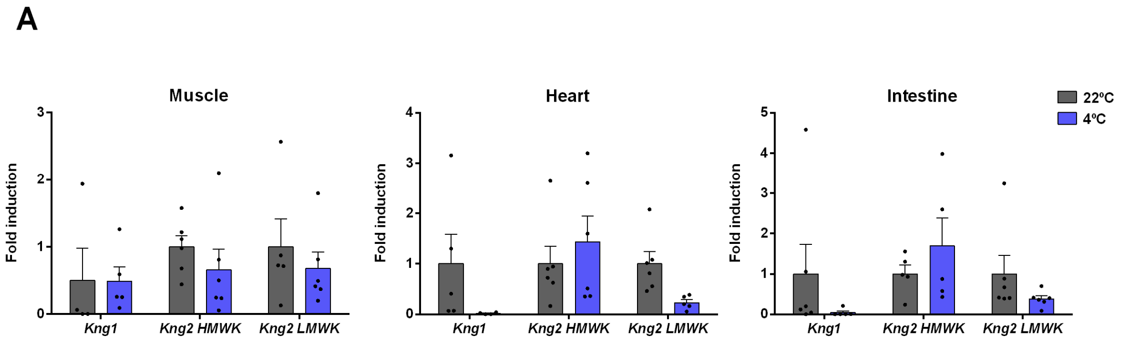

Supplementary figure 1: Expression of Kng genes in different tissues during cold exposure. A) mRNA expression of the different Kngs in muscle, heart, and intestine of 3 months old C57BL/6J mice (n=6 animals). Data are presented as means ± s.e.m. (bars); two-tailed unpaired Student's t-test. Source data are provided as a Source Data file.

Supplementary Figure 2

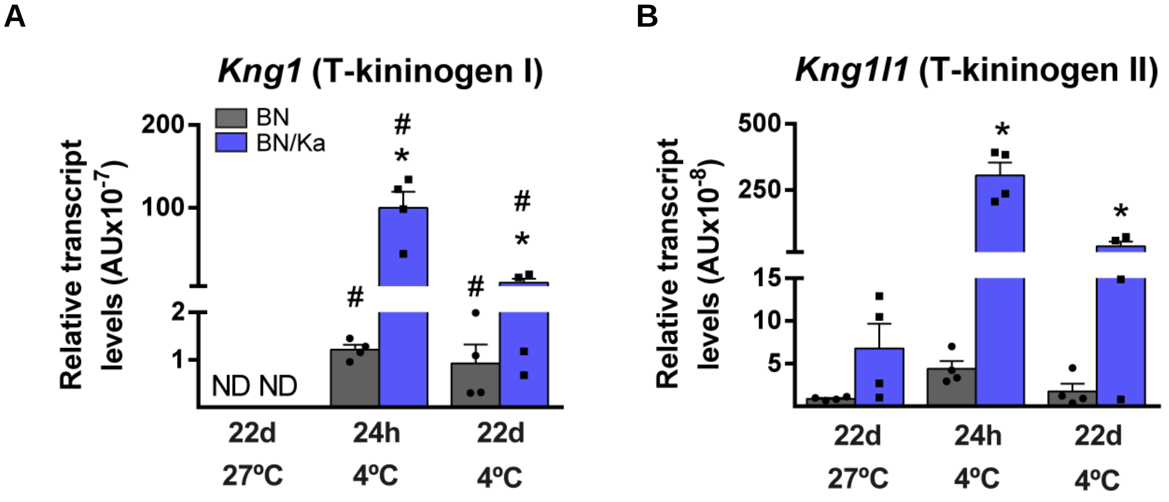

Supplementary figure 2: Expression of Kngs in iBAT of BN/Ka rats under temperature challenges. **A**) mRNA expression of T-kininogen I (*Kng1*) and **B**) T-kininogen II (*Kng1/1*) in iBAT from 12 weeks old WT and BN/Ka rats under thermoneutral (Tn) conditions and after 24-hours or 22-days exposure to cold (4°C) (n=4 animals). ND, not detectable. Data are presented as means ± s.e.m. (bars). *$P < 0.05$ versus BN; $^{\#}P < 0.05$ versus thermoneutrality. *P*-values determined by two-tailed unpaired Student's t-test (for genotypes) and two ways ANOVA with Tukey's post hoc test (for temperature) (**A, B**). Source data are provided as a Source Data file.

Supplementary Figure 3

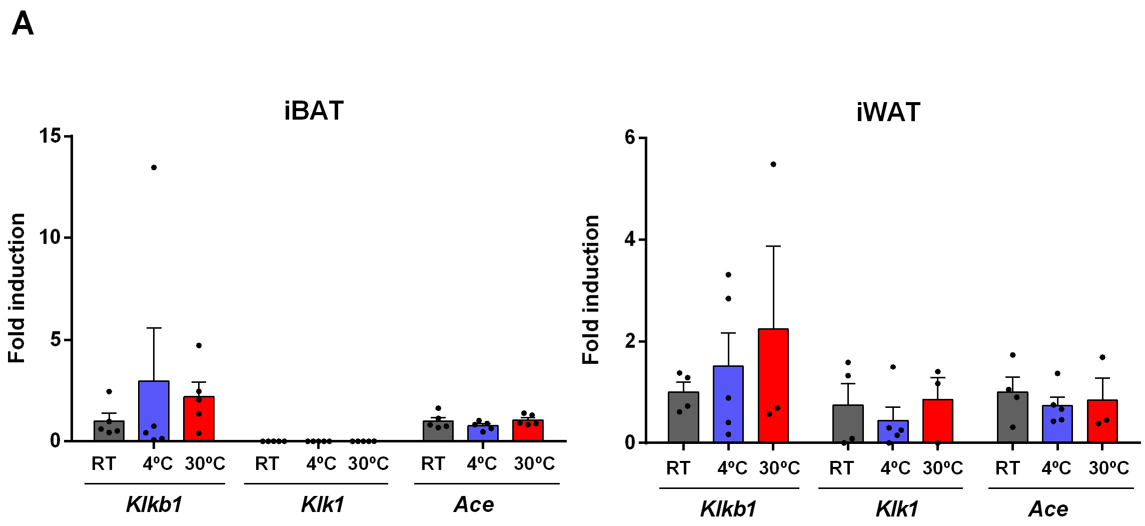

Supplementary figure 3: Expression of other components of the kinin system in iBAT after thermogenic challenges. A) mRNA expression of plasma kallikrein b1 (Klkb1), kininase 1 (Klk1) and angiotensin-converting enzyme (Ace) in iBAT and iWAT after 1 week exposition to 4ºC or 30ºC (n=5 animals, except for iWAT 30ºC condition where n=3 and iWAT RT where n=4). Data are presented as means ± s.e.m. (bars); one way ANOVA with Tukey's post hoc test. Source data are provided as a Source Data file.

Supplementary Figure 4

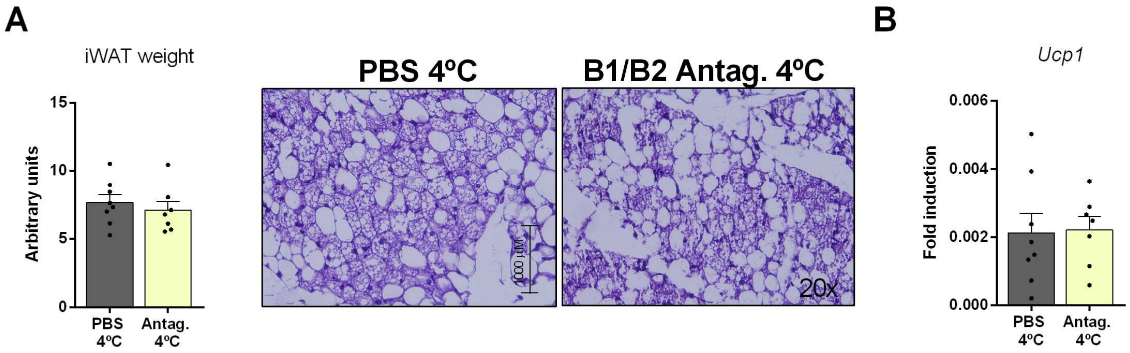

Supplementary figure 4: Effects of local pharmacological inhibition of B1/B2 receptors on iBAT. A) iWAT weight normalized to tibia length of WT mice exposed for 1 week to 4°C and then implanted in the back with mini-pumps delivering either PBS or a cocktail of B1 and B2 antagonists (n=8 animals for PBS and n=7 animals for antagonists); H&E-stained histological sections of iWAT. B) mRNA expression of Ucp1 in iWAT (n=8 animals for PBS and n=7 animals for antagonists). Data are presented as means ± s.e.m. (bars); two-tailed unpaired Student's t-test. Source data are provided as a Source Data file.

Supplementary Figure 5

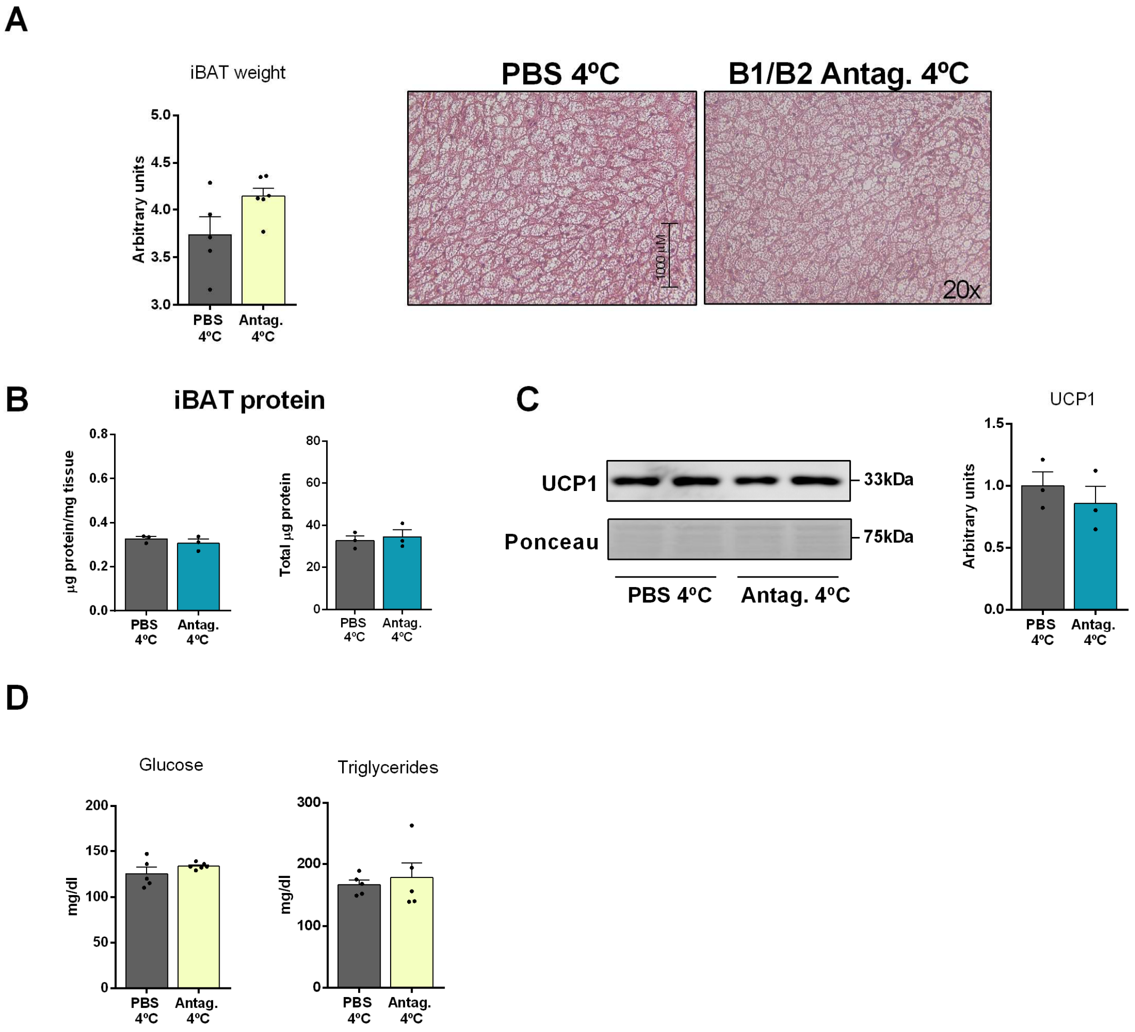

Supplementary figure 5: Effects of local pharmacological inhibition of B1/B2 receptors on iWAT. A) iBAT weight normalized to tibia length of WT mice exposed for 1 week to 4°C and then implanted in the leg with mini-pumps delivering either PBS or a cocktail of B1 and B2 antagonists (n=6 animals); H&E-stained histological sections of iBAT. B) Quantification of total protein per milligram of tissue and in the entire iBAT depot (n=3 animals). C) Expression and quantification of UCP1 protein (n=3 animals). D) Levels of glucose and triglycerides (n=6 animals). Data are presented as means ± s.e.m. (bars); two-tailed unpaired Student's t-test. Source data are provided as a Source Data file.

Supplementary Figure 6

A

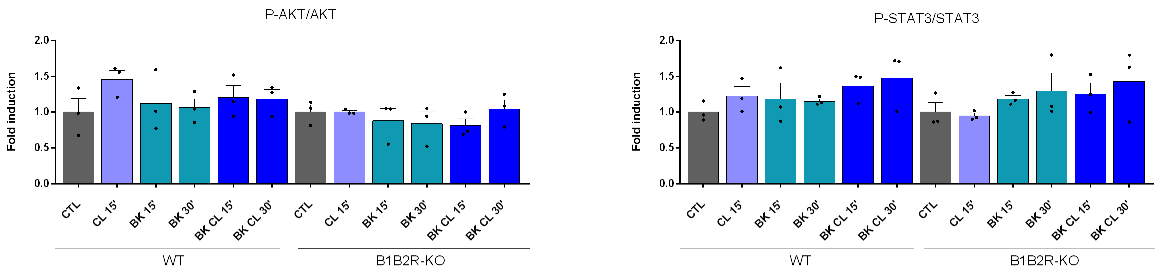

Supplementary figure 6: Pathways involved in the regulation of thermogenesis by the Kallikrein-kinin system. A) Phosphorylation status of AKT and STAT3 in primary WT and B1B2R-KO brown adipocytes exposed to CL316243, bradykinin or both for 15 or 30 min (n=3 independent cell culture experiments), normalized with total AKT and STAT3. Data are presented as means ± s.e.m. (bars); one way ANOVA with Tukey's post hoc test. Source data are provided as a Source Data file.

Supplementary Figure 7

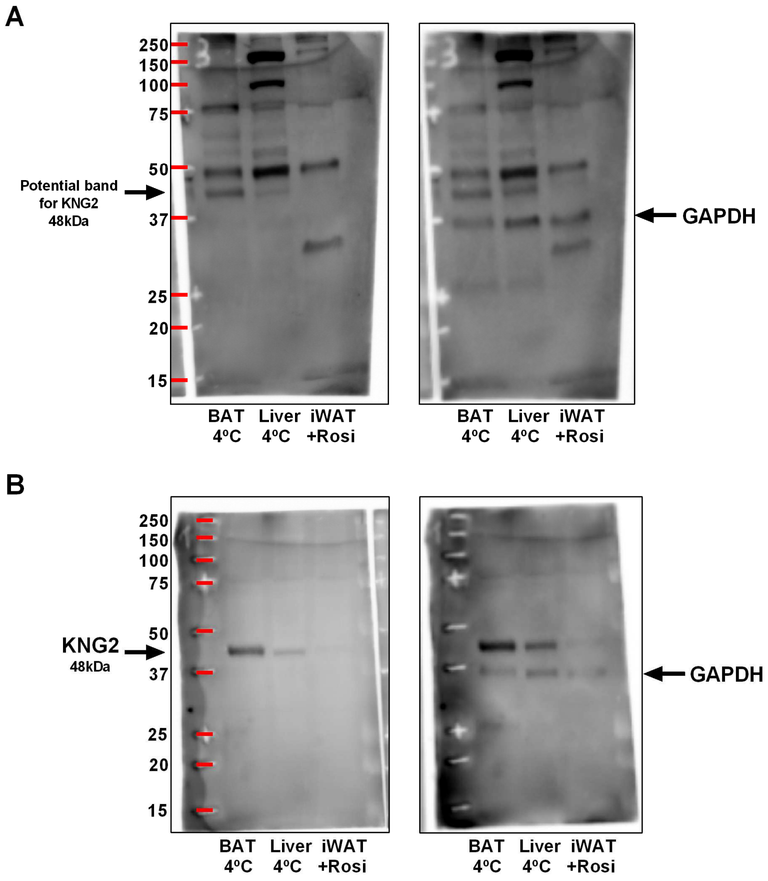

Supplementary Figure 7: Purification of KNG2 antibody. **A**) Western blot representing the profile of immunoreactivity of the crude anti-serum obtained in rabbits immunized against the specific KNG2 peptide C-Ahx-LYRVTKRAKMDGSAT-amide. The right panel corresponds to re-immunoblotting with the GAPDH antibody to show loading. **B**) Western blot with same tissue extracts using the specific peptide-based affinity-purified antibody. Evidence of a single band detection corresponding to the KNG2 size at 48 kDa. Relative intensity of the band in tissues is fully concordant with the *Kng2* mRNA expression in the tissue conditions quoted in Fig 1. The right panel corresponds to re-immunoblotting with the GAPDH antibody to show loading.

Supplementary Tables

Supplementary table 1: General and circulating parameters in BN/Ka rats.

	27ºC	
	**BN (n=4)**	**BN/Ka (n=4)**
Body weight (g)	274.3 ± 1.8	274.0 ± 1.5
Food consumption (g/day/animal)	24.8 ± 1.2	26.5 ± 2.9
Tissue weight<br>*(mg of tissue/Body weight, g)*		
iBAT	0.86 ± 0.11	**1.28 ± 0.06***
eWAT	2.93 ± 0.35	2.84 ± 0.69
Liver	29.13 ± 0.39	27.55 ± 2.81
Circulating parameters		
Glucose (mg/dl)	98.75 ± 2.06	**84.5 ± 3.01****
Insulin (ng/ml)	0.77 ± 0.08	0.94 ± 0.17
Leptin (ng/ml)	3.20 ± 0.57	3.52 ± 0.99

12 weeks old BN and BN/Ka rats exposed to thermoneutrality (27ºC) for 22 days. All measurements are means ± s.e.m. Data were analyzed by two tailed unpaired Student's t-test (*P<0.05 when compared with BN).

Supplementary table 2: Circulating parameters in mini-pumps inserted mice.

	PBS (n=3-8)	Antagonists (n=5-7)
Glucose (mg/dl)	150±6	167±10
Triglycerides (mg/dl)	170±6	157±12
Insulin (pg/ml)	1226±143	1041±69
Leptin (pg/ml)	609±132	960±166
PAI1 total (pg/ml)	1107±223	1479±170
Resistin (pg/ml)	1347±494	2198±145
Il-6 (pg/ml)	13,1±3,7	10,6±3,9

9 weeks old mice were inserted subcutaneously with a mini-pump diffusing either PBS or a cocktail of B1 and B2 antagonists upon iBAT localization and then exposed to cold (4ºC) for 1 week. All measurements are means ± s.e.m. Data were analyzed by two tailed unpaired Student's t-test.

Supplementary table 3: General and circulating parameters in B1B2R-KO mice after temperature challenges.

	22ºC		4ºC		30ºC	
	**WT (n=7)**	**B1B2R-KO (n=7)**	**WT (n=8)**	**B1B2R-KO (n=8)**	**WT (n=5)**	**B1B2R-KO (n=6)**
Body weight (g)	25,9±0,7	26,6±0,9	29,5±0,3$^{\#\#\&}$	28,4±0,7$^{\#}$	32±0,6$^{\#\#\#\&\&\&}$	30,2±1,1$^{\#\#\&}$
Food consumption (g/day/animal)	3,1±0,2	3,2±0,1	8,1±0,1$^{\#\#\#\&\&\&}$	**6,2±0,2$^{***\#\#\#\&\&\&}$**	2,6±0,1$^{\#\#\&\&\&}$	**2,9±0,1****
Tibia length (mm)	17,2±0,04	17,1±0,2	17,4±0,04	17,1±0,04	17,6±0,04$^{\#\&\&}$	17,3±0,1
Tissue weight *(mg of tissue/tibia length, mm)*						
iBAT	3,5±0,2	3,4±0,2	6,6±0,3$^{\#\#\#\&\&\&}$	6±0,2$^{\#\#\#\&\&\&}$	5,5±0,6$^{\#\#\&\&\&}$	**3,2±0,3*****
eWAT	20,1±1,03	23,2±1,5	12,5±1,1$^{\#\#\&\&\&}$	**19,3±1,2****	40,9±5,6$^{\#\#\#\&\&}$	**20,3±2,6*****
iWAT	11,9±1,6	12,1±0,5	9,4±0,4	**11,8±0,7***	21,2±2,2$^{\#\#\&\&}$	**13,1±1,4****
mWAT	9,3±1,1	10,7±1,1	5,6±0,5$^{\#\#\&\&}$	**8,5±1,1***	15,6±1,9$^{\#\#}$	**7,3±0,7*****
Liver	62,7±3	**74,9±2,1****	90,9±1,4$^{\#\#\#\&\&\&}$	85,7±2,7$^{\#\#\#\&}$	77,3±3,5$^{\#}$	77±5,5$^{\#}$
Heart	7,3±0,5	7,3±0,2	11±0,3$^{\#\#\#\&\&\&}$	10,5±0,3$^{\#\#\#\&\&\&}$	7,5±0,3	8,2±0,4
Circulating parameters						
Glucose (mg/dl)	157±8,4	182±7,6	152±3,2	**185±6,2***	154±3,6	166±8,6
Triglycerides (mg/dl)	195±10,7	200±11,2	223±8,1	200±19,2	246±11,7	208±15
Insulin (pg/ml)	751±120,5	835±97,7	708±119,6	603±122,8	801±159,3	754±130,9
Leptin (pg/ml)	1912±309,6	1846±383,6	**1116±89,3$^{\#}$**	1646±264,4	2565±782,3	**637±95,7***
PAI1 total (pg/ml)	450±119,6	943±232,5	1606±507,8	1075±134,9	698±99,6	1808±606,7$^{\#}$
Resistin (pg/ml)	913±42,4	1127±135,9	1010±87,2	851±68,2	450±90,8$^{\#\#\&\&\&}$	601±48$^{\&\&}$
Il-6 (pg/ml)	ND	ND	ND	ND	28,3±16,4	85,1±43,4
TNF-α (pg/ml)	ND	ND	ND	ND	22,7±6,6	10,3±1,4
Mcp-1 (pg/ml)	ND	ND	ND	ND	52,5±18,2	69,9±22,2
Adiponectin (μg/ml)	61,9±6,4	44,4±6,5	39,1±7,6	53,3±5,2	56,3±1,5	**42±4,1***

12 weeks old mice were exposed to cold (4ºC) or thermoneutrality (30ºC) for 1 week. iBAT, interscalpular BAT; iWAT, inguinal WAT; eWAT, epididymal WAT; mWAT, mesenteric WAT; ND, not-detectable. All measurements are means ± s.e.m. Data were analyzed by one-way ANOVA (*P<0.05, **P<0.01 and ***P<0.001 when compared with its own CTL; $^{\#}$P<0.05, ·$^{\#\#}$P<0.01 and $^{\#\#\#}$P<0.001 when compared with WT 22ºC; $^{\&}$P<0.05, $^{\&\&}$P<0.01 and $^{\&\&\&}$P<0.001 when compared with B1B2R-KO 22ºC).

Supplementary table 4: Transcript expression in iBAT from B1B2R-KO mice.

	22ºC		4ºC		30ºC	
	**WT (n=5)**	**B1B2R-KO (n=5)**	**WT (n=5)**	**B1B2R-KO (n=5)**	**WT (n=5)**	**B1B2R-KO (n=5)**
Thermogenesis						
Ucp1	1±0,1	1±0,3	2,3±0,2$^{\#\#\#\&\&\&}$	2,4±0,2$^{\#\#\#\&\&\&}$	1,04±0,1	3,7±0,8$^{\#\#\&\&}$
Dio2	1±0,2	2±0,6	3,6±0,9$^{\#}$	2,7±0,4	1,5±0,3	3,3±1,8
Prdm16	1±0,2	0,9±0,2	0,5±0,03$^{\#}$	0,7±0,1	2,3±0,2$^{\#\#\&\&}$	3,1±0,3$^{\#\#\#\&\&\&}$
Pgc1a	1±0,2	1,9±0,5	1,4±0,3	1,3±0,2	3,2±0,3$^{\#\#}$	3,3±0,5$^{\#\#}$
Cidea	1±0,3	0,7±0,1	0,6±0,1	0,4±0,04	1,8±0,1$^{\&\&}$	**2,6±0,2*$^{\#\#\#\&\&\&}$**
Cebpb	1±0,3	0,6±0,2	1,7±0,6	0,6±0,1	1,2±0,5	2,5±1,8
Leptin	1±0,3	1±0,2	0,7±0,2	0,5±0,1	16,3±2,04$^{\#\#\#\&\&\&}$	**6,04±1,6*****
Kng2 HMWK	1±0,2	0,5±0,1	1,4±0,3	2,3±0,5$^{\#\&\&}$	1,1±0,2	0,9±0,2
Kng2 LMWK	1±0,1	1,1±0,1	3,1±0,5$^{\#\#\#\&\&\&}$	3,6±0,2$^{\#\#\#\&\&\&}$	1,9±0,1$^{\#\#\#\&\&}$	**1,2±0,2*$^{\#}$**
Fatty acid and glucose metabolism						
Lpl	1±0,1	0,8±0,1	1,1±0,1	1,1±0,1		
Fabp4	1±0,1	0,9±0,1	0,9±0,2	0,8±0,02		
Fas	1±0,2	0,7±0,1	0,8±0,3	0,8±0,1		
Cd36	1±0,2	1,2±0,2	1,2±0,4	1,1±0,1		
Dgat	1±0,2	0,8±0,2	1±0,4	1±0,1		
Lcad	1±0,2	0,8±0,1	0,9±0,2	0,9±0,1		
Acox1	1±0,1	0,8±0,1	0,8±0,1	1,1±0,1		
Glut1	1±0,1	0,8±0,1	0,9±0,1	0,7±0,1		
Glut4	1±0,1	**0,7±0,1***	0,8±0,2	0,7±0,1		
Angiogenesis						
Angiopt1	1±0,2	1,2±0,1	0,2±0,02$^{\#\#\#\&\&\&}$	0,2±0,01$^{\#\#\#\&\&\&}$		
Cd31	1±0,1	0,8±0,1	0,9±0,1	0,8±0,1		
Flt1	1±0,1	1±0,1	0,7±0,1	0,6±0,1$^{\#}$		
Kdr	1±0,2	0,9±0,1	0,9±0,1	0,7±0,1		
Vegfa	1±0,2	0,8±0,1	0,8±0,1	0,8±0,1		
Immune						
Tnf	1±0,2	1,1±0,03	1,3±0,2	1,2±0,1		
Nos2	1±0,2	0,7±0,1	0,4±0,1$^{\#\#}$	0,4±0,1$^{\#\#}$		
Ccl2	1±0,2	3,4±1,6	3,1±0,7	2,2±0,1		
Il6	1±0,1	1,4±0,2	1,9±0,6	1,1±0,1		
Arg1	1±0,3	5,7±2,8	3±2,2	8,6±6,3		
Mrc1	1±0,1	1,1±0,1	1,2±0,3	0,8±0,1		
Clec10a	1±0,1	1,1±0,1	1,8±0,6	0,7±0,1		

12 weeks old mice were exposed to cold (4ºC) or thermoneutrality (30ºC) for 1 week. All measurements are means ± s.e.m. Data were analyzed by one-way ANOVA (*P<0.05 and ***P<0.001 when compared with its own CTL; $^{\#}$P<0.05, ·$^{\#\#}$P<0.01 and $^{\#\#\#}$P<0.001 when compared with WT 22ºC; $^{\&\&}$P<0.01 and $^{\&\&\&}$P<0.001 when compared with B1B2R-KO 22ºC).

Supplementary table 5: Transcript expression in iWAT from B1B2R-KO mice.

	22ºC		4ºC		30ºC	
	**WT (n=5)**	**B1B2R-KO (n=5)**	**WT (n=5)**	**B1B2R-KO (n=5)**	**WT (n=4)**	**B1B2R-KO (n=5)**
Thermogenesis						
Ucp1	1±0,4	1,6±1,1	73,3±25,3$^{\#\&}$	45,3±19,6	0,01±0,01	0,04±0,02
Dio2	1±0,4	1,2±0,8	21±6,1$^{\#\#\&\&}$	**7,7±2,2***	0,1±0,01	0,5±0,2
Prdm16	1±0,2	0,7±0,1	0,8±0,1	1,1±0,1	1,3±0,4	0,8±0,1
Pgc1a	1±0,2	0,9±0,3	2,2±0,4$^{\#\&}$	2,5±0,2$^{\#\&\&}$	1,03±0,3	0,7±0,1
Cidea	1±0,4	0,9±0,2	3,6±0,9$^{\&}$	2,3±0,8	0,1±0,01	0,2±0,1
Cebpb	1±0,4	0,4±0,2	4,4±0,6$^{\#\#\#\&\&\&}$	**0,7±0,2*****	0,5±0,1	0,3±0,1
Leptin	1±0,2	0,6±0,2	0,6±0,1	1,2±0,5	1,4±0,7	0,2±0,1
Adipoq	1±0,2	0,7±,02	0,5±0,1	0,6±0,2	0,8±0,3	0,4±0,1
Kng2 HMWK	1±0,4	1,8±0,4	4,6±1,3$^{\#}$	2,8±0,3	8,3±3,8	39,8±24
Kng2 LMWK	1±0,4	1,3±0,4	66,7±29$^{\#\&}$	9,4±3,3	1,4±1	0,2±0,1
Fatty acid and glucose metabolism						
Lpl	1±0,2	0,7±0,2	1,1±0,3	1,1±0,2	1,1±0,5	0,7±0,09
Fabp4	1±0,2	1±0,2	1,2±0,2	1,5±0,3	0,9±0,3	0,5±0,1
Fas	1±0,3	0,9±0,2	2,2±0,4$^{\#\&}$	2±0,3	0,3±0,1	0,7±0,2
Cd36	1±0,2	1±0,2	0,8±0,2	1,1±0,2	2,1±0,8	1,2±0,2
Mcad	1±0,2	0,7±0,1	2,3±0,4$^{\#\&\&}$	2,3±0,2$^{\#\&\&}$	0,8±0,3	0,6±0,1
Lcad	1±0,1	1±0,2	3,2±0,6$^{\#\#\&\&}$	3,2±0,4$^{\#\#\&\&}$	0,6±0,1	0,6±0,1
Acox1	1±0,2	1±0,2	1,7±0,4	1,9±0,3	0,8±0,3	0,5±0,1
Glut1	1±0,2	0,9±0,2	0,8±0,2	0,9±0,3	0,6±0,1	0,8±0,1
Glut4	1±0,1	1±0,2	2,5±0,4$^{\#\&}$	2,2±0,3	1,3±0,5	1,1±0,3
Angiogenesis						
Angiopt1	1±0,4	0,4±0,1	0,5±0,1	0,7±0,2	2,2±0,8$^{\&}$	1,5±0,3
Cd31	1±0,2	0,6±0,1	0,5±0,1	0,8±0,1	1,8±0,1$^{\#\&\&}$	1,9±0,1$^{\#\&\&}$
Flt1	1±0,3	0,6±0,1	0,4±0,1	0,6±0,2	1,4±0,5	1,4±0,2
Kdr	1±0,2	0,7±0,2	0,7±0,2	0,9±0,2	0,7±0,2	0,5±0,04
Vegfa	1±0,4	1,1±0,3	0,6±0,1	1,1±0,3	1,3±0,5	0,9±0,1
Immune						
Tnf	1±0,7	1,2±0,6	0,4±0,2	0,7±0,5	2,5±0,9	3,2±0,5
Nos2	1±0,3	0,7±0,2	0,5±0,1	0,8±0,3	1±0,1	0,6±0,1
Ccl2	1±0,5	3,3±1,2	1,1±0,5	0,8±0,3	2±0,2	4,1±0,9
Il6	1±0,4	1,4±0,5	0,7±0,3	0,9±0,3	3±0,5	5,3±0,9$^{\#\#\&\&}$
Arg1	1±0,3	10,1±7,6	2,7±2,1	0,9±0,3	0,4±0,1	3±1,2
Mrc1	1±0,2	1,5±0,5	0,3±0,1	0,7±0,3	1,7±0,4	2,8±0,5$^{\#}$
Clec10a	1±0,2	1,1±0,2	0,4±0,1	0,8±0,3	2,1±0,4	3,2±0,6$^{\#\#\&\&}$

12 weeks old mice were exposed to cold (4ºC) or thermoneutrality (30ºC) for 1 week. All measurements are means ± s.e.m. Data were analyzed by one-way ANOVA (*P<0.05 and ***P<0.001 when compared with its own CTL; $^{\#}$P<0.05, $^{\#\#}$P<0.01 and $^{\#\#\#}$P<0.001 when compared with WT 22ºC; $^{\&}$P<0.05, $^{\&\&}$P<0.01 and $^{\&\&\&}$P<0.001 when compared with B1B2R-KO 22ºC).
