# Supplementary material for: The kallikrein–kinin pathway as a mechanism for auto-control of brown adipose tissue activity
Source: Nat Commun. 2020 May 1;11:2132. doi: 10.1038/s41467-020-16009-x (PMC7195474; doi:10.1038/s41467-020-16009-x)
Supplement: Supplementary file 3 — Reporting Summary [file 41467_2020_16009_MOESM3_ESM.pdf]

nature research

Corresponding author(s): Dr. Francesc Villarroya

Last updated by author(s): Mar 27, 2020

Reporting Summary

Nature Research wishes to improve the reproducibility of the work that we publish. This form provides structure for consistency and transparency in reporting. For further information on Nature Research policies, see Authors & Referees and the Editorial Policy Checklist .

Statistics

For all statistical analyses, confirm that the following items are present in the figure legend, table legend, main text, or Methods section.

n/a	Confirmed	
☐	☒	The exact sample size (*n*) for each experimental group/condition, given as a discrete number and unit of measurement
☒	☐	A statement on whether measurements were taken from distinct samples or whether the same sample was measured repeatedly
☐	☒	The statistical test(s) used AND whether they are one- or two-sided<br>*Only common tests should be described solely by name; describe more complex techniques in the Methods section.*
☒	☐	A description of all covariates tested
☒	☐	A description of any assumptions or corrections, such as tests of normality and adjustment for multiple comparisons
☐	☒	A full description of the statistical parameters including central tendency (e.g. means) or other basic estimates (e.g. regression coefficient) AND variation (e.g. standard deviation) or associated estimates of uncertainty (e.g. confidence intervals)
☐	☒	For null hypothesis testing, the test statistic (e.g. *F*, *t*, *r*) with confidence intervals, effect sizes, degrees of freedom and *P* value noted<br>*Give P values as exact values whenever suitable.*
☒	☐	For Bayesian analysis, information on the choice of priors and Markov chain Monte Carlo settings
☒	☐	For hierarchical and complex designs, identification of the appropriate level for tests and full reporting of outcomes
☒	☐	Estimates of effect sizes (e.g. Cohen's *d*, Pearson's *r*), indicating how they were calculated

Our web collection on statistics for biologists contains articles on many of the points above.

Software and code

Policy information about availability of computer code

Data collection	No software was used for data collection.
Data analysis	Thermographic pictures analysis: QuickReport 1.2 SP 2 software FLIR Systems<br>Immunofluorescence and H&E pictures analysis: ImageJ 2.0 NIH and Adiposoft extension<br>Quantification western blot: Multi Gauge V3.0 Software Fujifilm, Tokyo, Japan<br>Data analysis: GraphPad Prism 6 Software Inc., San Diego, CA, USA

For manuscripts utilizing custom algorithms or software that are central to the research but not yet described in published literature, software must be made available to editors/reviewers. We strongly encourage code deposition in a community repository (e.g. GitHub). See the Nature Research guidelines for submitting code & software for further information.

Data

Policy information about availability of data

All manuscripts must include a data availability statement. This statement should provide the following information, where applicable:

- Accession codes, unique identifiers, or web links for publicly available datasets
- A list of figures that have associated raw data
- A description of any restrictions on data availability

RNA-seq was performed as previously published (Quesada-Lopez T, et al Nat Commun 7, 13479 (2016)). The raw data are accessible in Gene Expression Omnibus (GEO) using accession number GSE77534. The complete list of cold-modulated genes in BAT revealed by analysis of RNA-seq data is available at http://lmedex.ulb.ac.be/data.php.

Field-specific reporting

Please select the one below that is the best fit for your research. If you are not sure, read the appropriate sections before making your selection.

☒ Life sciences ☐ Behavioural & social sciences ☐ Ecological, evolutionary & environmental sciences

For a reference copy of the document with all sections, see nature.com/documents/nr-reporting-summary-flat.pdf

Life sciences study design

All studies must disclose on these points even when the disclosure is negative.

Sample size	The minimum of animals allowing statistical significance has been proposed and accepted by ethical committees, based on previous studies published in the same field. (Quesada-Lopez et al, 2016 Nature Comms; Cereijo et al 2018 Cell metab)
Data exclusions	Outliers were determined with graphpad software. Some were detected in the cold experiment settings and excluded (1 animal has been excluded on 29). Indeed, one WT animal in RT condition behaved differently from the rest of the group. His weight was lower and he seamed weaker even though was the same age as the group. When graphpad detected several outliers while measuring different parameters of the animal, we took the decision to remove the whole animal data from the experiment. At the autopsy, this animal appeared to be sick (skin damages).
Replication	Every experiment was repeated at least 3 times to assess the reproducibility of the experimental findings. All attempts of replication were successful.
Randomization	After checking that there were no statistical differences in initial body weights, animals were randomly allocated to experimental groups.
Blinding	The management of mouse KO colonies and wild-type littermates as well as specificities of the treatment of cell cultures precluded a fully blinded assessment of these experiments. The quantitative analytical procedures (metabolite and hormonal determinations, qRT-PCR-based analyses, immunoblots, Multiplex assays) were performed in a blinded way.

Reporting for specific materials, systems and methods

We require information from authors about some types of materials, experimental systems and methods used in many studies. Here, indicate whether each material, system or method listed is relevant to your study. If you are not sure if a list item applies to your research, read the appropriate section before selecting a response.

Materials & experimental systems

n/a	Involved in the study	
☐	☒	Antibodies
☒	☐	Eukaryotic cell lines
☒	☐	Palaeontology
☐	☒	Animals and other organisms
☒	☐	Human research participants
☒	☐	Clinical data

Methods

n/a	Involved in the study	
☒	☐	ChIP-seq
☒	☐	Flow cytometry
☒	☐	MRI-based neuroimaging

Antibodies

Antibodies used	UCP-1 Rabbit Abcam (Cambridge, UK) ab10983 lot: GR3215381-2<br>KNG1 Rabbit Abcam (Cambridge, UK) ab175386<br>KNG2 Rabbit AntibodyBcn (Barcelona, Spain) 8317<br>TH Rabbit Millipore (Madrid, Spain) ab152 lot: 2639432<br>β-actin Mouse Sigma Aldrich (Madrid, Spain) #A5441<br>HRP-conjugated anti-rabbit Goat Abcam (Cambridge, UK) ab6721<br>HRP-conjugated anti-mouse Goat Bio-rad (Madrid, Spain) #1721011<br>AlexaFluor 488-conjugated anti-rabbit Goat Thermo Fisher Scientific A-11034
Validation	-UCP1 antibody validated by manufacturer Abcam in WB, IHC, ICC/IF and tested in Mouse, Rat, Spermophilus tridecemlineatus. Cited in 255 publications.<br>-Kng1 antibody validated by manufacturer Abcam in WB and IHC-P and tested in Mouse, Rat and Human. Cited in one publication: Wu Z et al. Int J Mol Sci 17:N/A (2016).<br>-Kng2 antibody validated with different mice tissues (Spleen, Liver, iWAT, BAT) in different thermogenic conditions. A western blot of the bleeding chosen before and after purification of the antibody is shown in supplementary figure 7.<br>-TH antibody routinely evaluated by Western Blot on PC12 lysates by manufacturer Millipore. Cited in 678 publications.

-β-actin antibody validated by manufacturer using cultured human or chicken fibroblast cell extracts in WB, IHC-P, IF, ELISA. Reactive in pig, Hirudo medicinalis, bovine, rat, canine, feline, human, rabbit, carp, mouse, guinea pig, chicken, sheep. Cited in 5335 publications.

Animals and other organisms

Policy information about [studies involving animals](); [ARRIVE guidelines]() recommended for reporting animal research

Laboratory animals	3 months old males C57BL/6-Bdkrb2/Bdkrb1tm1Mki/J (B1B2R-KO) mice (Jackson laboratory Bar Harbor, ME, USA) and their littermate controls (WT), 3 weeks old males Swiss and C57BL/6J mice (Harlan Laboratory Indianapolis, IN, USA), as well as 3 months old males Brown Norway rats (BN/RijHsd colony) (Harlan) and BN/Ka breeder rats (provided by Dr E. Kaschina and T.Unger Charité University, Berlin, Germany) were used for this study.
Wild animals	The study did not involve wild animals.
Field-collected samples	No field collected samples were used in the study.
Ethics oversight	All experiments were performed in accordance with European Community Council directive 86/609/EEC, and experimental protocols as well as the number of animals, determined based on the expected effects size, were approved by the Institutional Animal Care and Use Committee at the University of Barcelona

Note that full information on the approval of the study protocol must also be provided in the manuscript.
